# Stretching Exercise for the Prevention of Postoperative Neck Symptoms Following Thyroid Surgery

**DOI:** 10.1089/ve.2021.0003

**Published:** 2021-09-30

**Authors:** Akira Miyauchi, Yasuhiro Ito, Akihiro Miya

**Affiliations:** Department of Surgery, Kuma Hospital, Hyogo, Japan.

**Keywords:** thyroid surgery, neck complaints, neck discomfort symptoms, stretching exercise

## Abstract

**Introduction:**

Thyroid surgery is a popular and effective treatment for many thyroid diseases, including thyroid cancer. However, patients who undergo thyroid surgery often experience discomfort symptoms such as stretching, pressing, or choking feelings in the neck, headache, shoulder stiffness, and difficulty in moving the neck or shoulders. These symptoms may persist for long durations. Based on our prospective randomized study published in 2005,^1^ we instructed patients who underwent thyroid surgery at Kuma Hospital to perform stretching exercises starting from the day after the surgery. Although we are of the opinion that this management is easy and effective, it might not be well recognized in the world. The purpose of this video is to demonstrate our method of instructing exercises to patients after thyroid surgery and to briefly describe our rehabilitation study.

**Materials and Methods:**

Patients who underwent thyroid surgery, except for those who had undergone reconstruction of the recurrent laryngeal nerve or trachea, were instructed stretching exercises starting the morning after surgery, to be performed at least thrice a day until their neck–shoulder symptoms disappeared. In our prospective randomized study, a total of 409 patients, including 234 patients with thyroid cancer, were randomly allocated into a stretching group and a control group that was not given the stretching instruction.^1^ Questionnaire surveys on neck symptoms were administered before the surgery and 1 week, 1 month, 6 months, and 1 year after the surgery.

**Results:**

The postoperative neck symptoms declined gradually after the surgery in both the groups over the study period. The total symptom scores were significantly lower in the stretching group than those in the control group at all the time points after the surgery (*p* < 0.0001).^1^ The difference between the total symptom score at 1 year and that before surgery decreased to 0.3 in the stretching group, suggesting nearly full recovery, whereas the difference was 1.8 in the control group, indicating persistence of the neck discomfort symptoms (*p* < 0.0001).^1^ Since we initiated the routine stretching exercise after thyroid surgery, we noticed in daily clinical practice that the proportion of patients who complained of postoperative neck symptoms had decreased and so did the extent of the symptoms. In the literature, there are several reports on neck–shoulder symptoms after thyroid surgery. However, we found only three articles, including ours, written in English on the prevention or treatment of these symptoms. Lee et al.^2^ reported that wound massage was effective for managing neck discomfort and voice changes after thyroid surgery. We believe that stretching might be easier for patients. Genç et al.^3^ tried cervical kinesiotaping after thyroid surgery and reported that it was not effective for postoperative neck–shoulder symptoms, although it reduced the use of analgesics.

**Conclusions:**

Stretching exercises, starting from the day after thyroid surgery, are simple and easy to perform and effectively prevent postoperative neck discomfort symptoms.

**No competing financial interests exist.:**

Runtime of video: 10 mins 4 secs

## Graphical abstract

**Figure:**
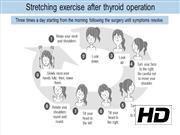

**Figure:**
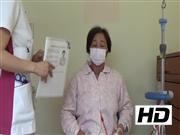

**Figure:**
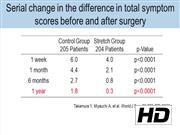

## Video

## Files

**MP4:** 

**PDF:** 
